# Glottic insufficiency caused by vocal fold atrophy with or without sulcus: systematic review of outcome measurements

**DOI:** 10.1007/s00405-024-08751-5

**Published:** 2024-07-18

**Authors:** Emke M. J. M. van den Broek, Stephanie D. Mes, Bas J. Heijnen, Antonius P. M. Langeveld, Peter Paul G. van Benthem, Elisabeth V. Sjögren

**Affiliations:** 1 Department of Otorhinolaryngology/Head and Neck Surgery, Leiden University Medical Center, Albinusdreef 2, PO-Box 9600, 2300 RC Leiden, The Netherlands; 2 Department of Otorhinolaryngology/Head and Neck Surgery, University Medical Center, Utrecht, The Netherlands

**Keywords:** Glottic insufficiency, Vocal fold atrophy, Sulcus, Scar, Treatment outcomes, Outcome measures, Core Outcome Set, Systematic review

## Abstract

**Purpose:**

Identifying outcome measurements instruments (OMIs) to evaluate treatment efficacy in patients with vocal fold atrophy and/or sulcus.

**Methods:**

Systematic review of records published before March 2021 by searching Pubmed and EMBASE. Included studies reported on adults (> 18 year) with dysphonia caused by glottic insufficiency due to vocal fold atrophy with or without sulcus, who were enrolled into a randomized controlled trial, a non-randomized controlled trial, a case-controlled study or a cohort study. All included studies described an intervention with at least one outcome measurement.

**Results:**

A total of 5456 studies were identified. After removing duplicates, screening title and abstract and full text screening of selected records, 34 publications were included in final analysis. From these 50 separate OMIs were recorded and categorized according to the ELS protocol by DeJonckere et al. (Eur Arch Otorhinolaryngol 258: 77–82, 2001). With most OMIs being used in multiple studies the total number of OMIs reported was 265. Nineteen (19) individual OMIs accounted for 80% of reports. The most frequently used OMIs according to category were: VHI and VHI-10 (subjective evaluation); G of GRBAS (perceptual evaluation); F0, Jitter and Shimmer (acoustic evaluation); MPT and MFR (aerodynamic evaluation) and glottic closure and mucosal wave (endoscopic evaluation). Of these OMIs VHI had a high percentage of significance of 90%.

**Conclusion:**

This systematic review identifies the most used OMIs in patients with glottic incompetency due to vocal fold atrophy and/or sulcus as a step toward defining a Core Outcome Set (COS) for this population.

**PROSPERO registration:**

238274.

## Introduction

Vocal fold atrophy, with or without sulcus, can lead to both reduced vocal fold closure and reduced vibration during phonation. There is however a great heterogeneity in presentation among patients, varying from mild to severe dysphonia with a similarly large variation in patient’s disease burden and findings during laryngostroboscopy. There is also a wide variation in treatment options for the glottic incompetence caused by these entities varying from speech language therapy (SLT) to different forms of laryngeal surgery. Vocal fold injection (VFI) with different injection material or laryngeal frame surgery (LFS) are often performed to improve glottic closure and for patients with sulcus there is also the option of microphonosurgery of the upper vibratory layers of the vocal fold using cold steel, lasers or tissue engineering techniques [2, 3]. Finally, there is also variation in outcome measurement instruments (OMIs) used to assess the severity of the condition and/or treatment outcome. These OMIs can be divided into subjective (self-assessment), perceptual, aerodynamic and acoustic measurements in addition to videolaryngostroboscopic findings according to the guidelines on voice quality assessment published and recently updated by the European Laryngological Society [1, 4].

Taking all of the above into account, evaluating treatment outcome in this patient group in a consistent way is challenging. To reliably evaluate and compare treatment outcomes it is important to formulate a core outcome set (COS). A COS is a consensus-based agreed minimum set of outcomes that should be evaluated and reported in clinical trials in a specific disease or population. For unilateral vocal fold paralysis (UVFP) such a COS has been formulated by Desuter et al., but up until now this is lacking for patients with non-paralytic glottic insufficiency [5, 6]. The protocol for developing a COS in an evidenced-based multi-step process has been described in the COSMIN guidelines (consensus-based standards for the selection of health measurement instruments) [7–9]. In the first step, a definition for the construct to be measured is created. In the second step, the existing OMIs for the defined construct are determined through a systematic review of the literature. In the third step, quality assessments of the included studies and the OMIs are performed. Finally, in the fourth step minimal outcome measures to be included in the COS are selected, often in a Delphi type procedure [10].

The definition of the construct of voice (step 1) has already been achieved through the work of the European Laryngological Society guidelines described earlier [1]. The aim of this review is to establish a systematic overview of the frequency and type of OMIs used in literature for patients with non-paralytic glottic insufficiency caused by vocal fold atrophy with or without sulcus as a further step towards formulating a COS for this patient population.

## Materials and methods

The design of this study was modeled on earlier studies such as by Desuter et al. in patients with vocal fold paralysis [5]. The construct to be measured in this review was determined to be “treatment effect in patients with dysphonia caused by vocal fold atrophy and/or sulcus”. A systematic review was conducted following the preferred reporting items for systematic reviews and meta-analyses (PRISMA) statement [11]. With assistance of a clinical librarian a search was performed in two databases Pubmed and EMBASE.

The search in Pubmed was constructed with following terms:

("Glottis"[MeSH Terms] OR "Glottis"[Title/Abstract] OR "glottic"[Title/Abstract] OR "glottal"[Title/Abstract] OR "vocal fold"[Title/Abstract] OR "vocal folds"[Title/Abstract] OR "vocal cord"[Title/Abstract] OR "vocal cords"[Title/Abstract]) AND ("incompetence"[Title/Abstract] OR "incompetency"[Title/Abstract] OR "insufficience"[Title/Abstract] OR "insufficiency"[Title/Abstract] OR "Atrophy"[MeSH Terms:noexp] OR "Atrophy"[Title/Abstract] OR "atrophic"[Title/Abstract] OR "Cicatrix"[MeSH Terms] OR "scar"[Title/Abstract] OR "scars"[Title/Abstract] OR "scarring"[Title/Abstract] OR "scarred"[Title/Abstract] OR "sulcus"[Title/Abstract] OR "paresis"[Title/Abstract] OR "pareses"[Title/Abstract] OR "hypomobil*"[Title/Abstract] OR "hypo mobil*"[Title/Abstract]).

The search in EMBASE was constructed with the following terms:

('glottis'/exp OR 'glottis' OR 'glottis':ti,ab,kw OR 'glottic':ti,ab,kw OR 'glottal':ti,ab,kw OR 'vocal fold':ti,ab,kw OR 'vocal folds':ti,ab,kw OR 'vocal cord':ti,ab,kw OR 'vocal cords':ti,ab,kw) AND ('incompetence':ti,ab,kw OR 'incompetency':ti,ab,kw OR 'insufficience':ti,ab,kw OR 'insufficiency':ti,ab,kw OR 'atrophy'/exp OR 'atrophy' OR 'atrophy':ti,ab,kw OR 'atrophic':ti,ab,kw OR 'scar'/exp OR 'scar':ti,ab,kw OR 'scars':ti,ab,kw OR 'scarring':ti,ab,kw OR 'scarred':ti,ab,kw OR 'sulcus':ti,ab,kw OR 'paresis':ti,ab,kw OR 'pareses':ti,ab,kw OR 'hypomobil*':ti,ab,kw OR 'hypo mobil*':ti,ab,kw).

Records published before March 2021 were identified. Included studies reported on adults (> 18 year) with dysphonia caused by glottic insufficiency due to vocal fold atrophy with or without sulcus, who were enrolled into a randomized controlled trial, a non-randomized controlled trial, a case-controlled study or a cohort study. All included studies described an intervention with at least one outcome measurement. Studies including other etiologies of glottic incompetence, with vocal fold atrophy and/or sulcus included as a subgroup, were not included. Pre-clinical studies, including animal and laboratory studies, were also excluded, as were case reports.

Title and abstract of the identified studies were screened by two independent reviewers (EB and SM). This was followed by full-text evaluation by one reviewer (EB). Baseline characteristics of the included studies were extracted (publication date, study type, diagnosis, number of patients, gender, mean age, treatment, follow up). Treatment was categorized in three groups: speech language therapy (SLT), surgery and regenerative therapy. The surgery group included microlaryngeal surgery (MLS) with cold steel instruments—labelled as “direct”- and with the use of laser—labelled as “laser”- and medialization technique vocal fold injection (VFI) and laryngeal framework surgery (LFS). All reported OMIs were extracted and listed according to frequency of use. Cumulative percentages were calculated to identify the OMIs accounting for 80% of the total of reported OMIs and outcomes were displayed in a Pareto diagram. Subsequently these OMIs were also subdivided in categories based on the ELS protocol for functional assessment of dysphonia [1] consisting of: subjective parameters, perceptual parameters, acoustic and aerodynamic parameters and videolaryngostroboscopic findings (henceforth endoscopic findings). In this last category we included the assessment of glottic closure and mucosal wave in accordance with a recent review on vocal fold scar [3]. OMIs on respiratory function were separately collected. OMIs that did not fit this category, nor the categories of the ELS protocol were reported as “additional” parameters. Finally, the percentage of significance for the most frequently used OMIs, defined as the number of studies with a significant post-treatment improvement divided by the total number of studies using this OMI, was calculated [5].

## Results

**Fig. 1:**
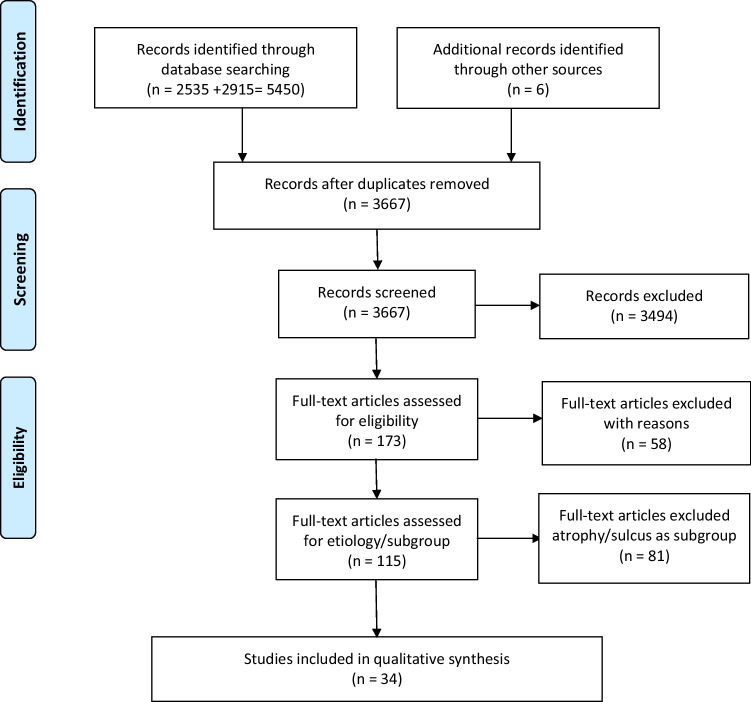
Flow chart of study inclusion process

A total of 173 articles were full text assessed. Fifty-eight (58) studies were excluded for the following reasons: population not clearly described (*n* = 11), no atrophy and/or sulcus included (*n* = 12), no therapy (*n* = 2), no relevant outcome (*n* = 2, complication, duration), no pre- and posttreatment outcome (*n* = 9), language other than English (*n* = 10, 1 Slovenic, 1 Polish, 1 Serbian, 2 Japanese, 2 Chinese, 3 Portuguese), abstract only (*n* = 10), duplicate (*n* = 2). Another 81 studies were excluded, because they included a mixed study populations of glottic insufficiencies including atrophy and/or sulcus but also scar, paresis, hypomobility, paralysis or other causes. Thirty-four (34) studies, only including atrophy and/or sulcus, were included in final qualitative synthesis (Fig. 1).

Table 1 shows the baseline characteristics and OMIs of the included studies [2, 12–44] .

**Table 1:** Overview included studies and their baseline characteristics

Reference		Study type	Diagnosis	Patients (*n*)	atrophy/sulcus/(control)	Gender (m:f)	Age (SD)	Treatment category	Treatment	Follow up
Bick [12]	2021	Cohort (retrospective)	atrophy	197	197/0	111:86	70.9 ± 10.15	SLT / surgery	SLT / SLT + surgery / surgery / no treatment	-
Desjardins [13]	2020	Clinical trial	atrophy	12	12/0	7:5	71.83 ± 7.76	SLT	VFE / VFE + IMST / VFE + EMST	1 m
Andreadis [14]	2020	Cohort (retrospective)	sulcus	13	0/13	5:8	28.9	surgery (direct)	MLS excision	6,3 m (mean)
Okui [15]	2020	Cohort (retrospective)	atrophy	53	53/0	41:12	69.0 ± 9.5	regenerative	VFI bFGF	6 m
Ma [16]	2020	Clinical trial	atrophy, sulcus,	21	7/8/(6)	15:6	64	regenerative	VFI ACF	12 m
Van den Broek [17]	2020	Cohort (retrospective)	atrophy, sulcus	29	14/15	12:17	50.5 ± 17.9	surgery	LFS bilateral medialization	12 m
Hu [18]	2019	Cohort (retrospective)	atrophy, sulcus	23	19/4	7:16	39.2	surgery	VFI autologous fat	6 m
Allensworth [19]	2019	Cohort (retrospective)	atrophy	21	21/0	17:4	76	surgery	VFI CaHA / LFS bilateral medialization	1.6 m (mean)
González-Herranz [20]	2019	Cohort (retrospective)	sulcus	10	0/10	-	-	surgery (direct)	MLS autologous fascia graft	6 m
Park [21]	2019	Cohort (retrospective)	sulcus	79	0/79	55:24	41	surgery (laser)	MLS laser (PDL/KTP)	6 m
Van den Broek [22]	2019	Cohort (retrospective)	atrophy, sulcus	24	15/9	0:24	39.5 ± 18.2	surgery	VFI autologous fat	12 m
Van den Broek [2]	2019	Cohort (retrospective)	atrophy, sulcus	68	30/38	18:50	40 ± 18.5	surgery	VFI HA	1 m
Miaśkiewicz [23]	2018	Cohort (retrospective)	sulcus	36	0/36	13:23	44.17 ± 11.95	surgery (combination)	MLS excison + VFI HA/CaHA	12 m
Kanazawa [24]	2018	Cohort (prospective)	sulcus	12	0/12	6:6	51.6	regenerative	VFI bFGF	3 m
Kaneko [25]	2015	Cohort (retrospective)	atrophy	22	22/0	13:3	72.9 ±	SLT	VFE	2 m
Miaśkiewicz [26]	2015	Cohort (retrospective)	sulcus	24	0/24	11:13	38.7 ±	surgery (direct)	MLS excision, subepithelial HZ(*)	8 m
Young [27]	2015	Cohort (retrospective)	atrophy	19	19/0	10:9	72 ± 11	surgery	VFI CMC; subsequent VFI autologous fat / VFI CaHA / LFS bilateral medialization	3 m
Hwang [28]	2013	Cohort (retrospective)	sulcus	25	25/0	17:8	37.6	surgery (laser)	MLS PDL	12 m
Yilmaz [29]	2012	Cohort (prospective)	sulcus	44	0/44	18:26	37	surgery (combination)	MLS excision, suture + VFI / LFS bilateral medialization	12 m
Hirano [30]	2012	Cohort (prospective)	atrophy	10	10/0	6:4	70.1 ± 5.3	regenerative	VFI bFGF	12 m
Gartner-Schmidt [31]	2011	Cohort (retrospective)	atrophy	275	275/0	133:142	71.8 ± 9.6	SLT / surgery	SLT / SLT + surgery / surgery / no treatment	-
Mau [32]	2010	Cohort (retrospective)	atrophy	67	67/0	33:34	71	SLT / surgery	SLT / SLT + surgery / surgery	-
Zhang [33]	2010	Cohort (prospective)	sulcus,	24	0/12/(12)	7:5	-	surgery (combination)	MLS gelatine sponge + VFI autologous fat	3 m
Pinto [34]	2007	Cohort (prospective)	sulcus	34	0/34	15:19	34.8	surgery (direct)	MLS autologous graft (fat or fascia)	12 m
Hsiung [35]	2006	Cohort (retrospective)	atrophy, sulcus	101	31/70	45:56	51.2	surgery (combination)	MLS autologous fat graft + VFI autologous fat	17 m (mean)
Tsunoda [36]	2005	Cohort (prospective)	sulcus	10	0/10	8:2	46.5	surgery (direct)	MLS autologous fascia graft	36 m
Hsiung [37]	2004	Cohort (retrospective)	sulcus	22	0/22	10:12	33.1	surgery (combination)	MLS autologous fascia graft + VFI autologous fat	16.6 m (mean)
Su [38]	2004	Cohort (retrospective)	atrophy, sulcus	27	17/10	16:11	41.5	surgery	LFS strap muscle transposition	4 m
Hsiung [39]	2003	Cohort (retrospective)	atrophy	21	21/0	9:12	46	surgery	VFI autologous fat	9.5 m (mean)
Chen [40]	2003	Cohort (retrospective)	atrophy, sulcus	24	16/8	-	-	surgery	VFI autologous fat	19.5 m (mean)
Hsiung [41]	2003	Cohort (retrospective)	atrophy, sulcus	16	14/2	7:9	51.3	surgery	VFI autologous fat	10 m (mean)
Ramig [42]	2001	Cohort (prospective)	atrophy	3	3/0	2:1	-	SLT	LSVT	-
Remarcle [43]	2000	Cohort (retrospective)	sulcus	45	0/45	7:38	36	surgery (laser, combination)	MLS CO2, collagen graft	5 m
Pontes [44]	1993	Cohort (retrospective)	sulcus	10	0/10	7:3	-	surgery (direct)	MLS slicing mucosa	-

*SLT* speech language therapy, *VFE* vocal function exercise, *IMST* inspiratory muscle strength training, *EMST* expiratory muscle strenght training,

*MLS* microlarygeal surgery, *VFI* vocal fold injection, *bFGF* basic fibroblast growth factor, *ACF* autologous cultured fibroblast, *LFS* laryngeal framework surgery, *CaHA* calciumhydroxylapatite, *PDL* pulsed dye laser, *KTP* potassium titanyl phosphate, *HA* hyaluronic acid, *CMC* carboxymethylcellulose, *LSVT* Lee Silverman voice therapy

(*) 2 patients adjuvant VFI HA

Two studies were randomized clinical trials [13, 16]; of which one was a double blind randomized controlled trial (RCT)[16]. All other reports were cohort studies of which 7 were prospective, 25 were retrospective; 11 studies included atrophy patients, 14 included sulcus, and 9 studies atrophy and sulcus. Two studies included a control group; one study with sulcus in a prospective cohort and one study with atrophy and sulcus in a RCT, mentioned above [16, 33].

In 3 studies treatment consisted of SLT, in 3 studies SLT or surgery, in 24 surgical treatments and in 4 regenerative therapy. The studies with surgical treatment were divided into “direct” microlaryngeal surgery (MLS), including microphonosurgical cold steel procedures invading subepithelial space with or without grafting (*n* = 6), MLS with laser surgery, including KTP, PDL and CO2 laser (*n* = 2), different types of medialization both VFI with different fillers (hyaluronic acid (HA), calciumhydroxylapatite (CaHA), carboxymethylcellulose (CMC)) and LFS (*n* = 10) or a combination of above described technics (*n* = 6). Follow-up varied from 1 to 36 months (median of 8 months). In Table 2 an overview is presented of the included studies and their OMIs.

**Table 2:** Overview included studies and their OMIs

Reference		OMI subjective	OMI perceptual	OMI acoustic	OMI aerodynamic	OMI endoscopic	OMI respiratory	OMI additional
Bick [12]	2021	VRQOL**, GFI**	GRBAS**	F0**, SPI	MPT**			
Desjardins [13]	2020	VHI-10, GFI, CPIB	CAPE-V (overall severity)	CPPS, NHR, APQ	MFR, Psub, AR, SPL	GC*, MW	MIP, MEP, FVC, FEV1, FEV1/FVC	
Andreadis [14]	2020	-	-			MW*		
Okui [15]	2020	VHI-30*	-	F0*, Jitt*, Shim*, HNR†, MR*	MPT*, MFR†			
Ma [16]	2020	VHI-30†, subjective rating (own questionnaire)† VAS**	G*			MW*		
Van den Broek [17]	2020	VHI-30*	G†	F0**, MR†	MPT†, DR†			
Hu [18]	2019	VHI-10*	G*B*R*A*S†	Jitt*, Shim†, NHR*	MPT†			
Allensworth [19]	2019	VHI-30*	CAPE-V*, G*		MPT*	GC*, MW*		
González-Herranz [20]	2019	VHI-10*	G*R*B† A†S*	Jitt†, Shim†, HNR†, MR	MPT*, s/z ratio*	GC, MW		
Park [21]	2019	VHI-10*	GRBAS*	F0†, Jitt*, Shim*, NHR*	MPT*, MFR†	GC, MW		
Van den Broek [22]	2019	VHI-30*	G†	F0†, MR†	MPT†, DR*			
Van den Broek [2]	2019	VHI-30*	-	F0†, MR*	MPT†, DR†			
Miaśkiewicz [23]	2018	VHI-30*	G*R*B*A*S*	F0†, Jitt†, RAP†, PPQ†, sPPQ†, vF0†, Shim*, APQ*, sAPQ†, vAm†, NHR†, SPI†		GC*, MW*		
Kanazawa [24]	2018	VHI-30*	-	F0*, Jitt†, Shim†, NHR*, MR*	MPT*, MFR†, SPL†			
Kaneko [25]	2015	VHI-10	GRBAS*	Jitt*, Shim†	MPT*, DR†, MFR†	GC*, MW*		
Miaśkiewicz [26]	2015	-	GR*B*AS*	F0, Jitt†, Jita*, PPQ, vF0, Shim†, ShdB†, APQ†, vAm†, NHR†, SPI†		GC†, MW†		
Young [27]	2015	VHI-10**	-	CPP, CSID	MFR, Psub, SPL,			
Hwang [28]	2013	VHI-30*	G*R*B*AS	F0*, Jitt*, Shim†, NHR†	MPT†, MFR*, Psub*	GC, MW*		QxM†, CFx*, CAx† (EEG)
Yilmaz [29]	2012	VHI-30*	G*R*B*A†S†	F0†, Jita*, RAP*, PPQ*, sPPQ*, vF0*, Shim*, APQ*, sAPQ*, vAm*, NHR*, MR*, SPI†, VTI*	MPT*, MFR*, resistance†, power†, efficiency*, pressure*	GC*, MW*		
Hirano [30]	2012	-	-	Jitt*, PPQ*, Shim*, APQ*, NHR*	MPT*, MFR**			
Gartner-Schmidt [31]	2011	VHI-10	-					
Mau [32]	2010	-	-					FCMs in NOMS*
Zhang [33]	2010	-	-	F0*, Jitt*, Shim*,NNE*	MPT*	GC, MW		
Pinto [34]	2007	subjective rating (5-point scale)*	subjective rating (5-point scale)*	F0†, Jitt†, Shim†		GC, MW		
Hsiung [35]	2006	subjective rating (3-point scale)**	subjective rating (3-point scale)	F0, Jitt, Shim, NHR	MPT			
Tsunoda [36]	2005	-	-		MPT*	GC, MW		
Hsiung [37]	2004	subjective rating (3-point scale)	G*R*B*	F0†, Jitt†, Shim†, HNR†	PT*	GC, MW*		
Su [38]	2004	-	G*R*B*A†S*, subjective rating (improvement yes/no)	F0†, Jitt*, Shim†, NHR†	MPT*, MFR*	GC, MW		
Hsiung [39]	2003	subjective rating (3-point scale)	G*R*B*	Jitt†, Shim†, HNR†	PT†	GC, MW*		
Chen [40]	2003	subjective rating (3-point scale)	G*R*B*	F0†, Jitt†, Shim†, HNR†	PT†	GC, MW*		
Hsiung [41]	2003	subjective rating (2-point scale)	GRB	Jitt, Shim, HNR	PT			MRI**
Ramig [42]	2001	-	CAPE-V (overall severity, loudness)	F0, MR	Psub, SPL	GC, MW		laryngeal EMG
Remarcle [43]	2000	subjective rating	-	F0†, own spectral analysis *	MPT*, DR†, PQ*	MW	VC	
Pontes [44]	1993	subjective rating (vocal fatique)	subjective rating (breathiness, harshness)	F0, own spectral analysis		GC, MW		

*VRQOL* voice related quality of life, *GFI* glottal function index, *VHI* voice handicap index, *CPIB* communicative participation item bank, *VAS* visual analog scale

*GRBAS* grade roughness breathiness asthenic strain, *CAPE-V* consensus auditory-perceptual evaluation of voice, *F0* fundamental frequency, *Jitt* jitter, *Jita* absolute jitter, *RAP* relative average perturbation, *PPQ* pitch perturbation quotient, *sPPQ* smoothed pitch perturbation quotient, *Shim* shimmer, *ShdB* absolute shimmer, *APQ* amplitude pertubation quotient, *sAPQ* smoothed amplitude pertubation quotient, *vAm* peak-to-peak amplitude, *NHR* noise to harmonic ratio, *HNR* harmonic to noise ratio, *SPI* soft phonation index, *VTI* voice turbulence index, *NNE* normalized noise energy, *MR* melodic range, *CPPS* smoothed cepstral peak prominence, *CPP* cepstral peak prominence, *CSID* cepstral spectral index of dysphonia, *MPT* maximum phonation time, *PT* phonation time, *PQ* phonation quotient, *DR* dynamic range, *MFR* mean flow rate, *Psub* subglottal pressure, *AR* aerodynamic resistance, *SPL* sound pressure level, *MV* mucosal wave, *GC* glottic closure, *MIP* maximal inspiratory pressure, *MEP* maximal expiratory pressure, *FVC* forced vital capacity, *FEV1* forced expiratory volume, *QxM* mean closed quotient, *CFx* irregularity of frequency, *CAx* irregularity of amplitude, *FCMs* functional communication measures, *NOMS* national outcomes measurement system, *MRI* magnetic resonance imaging, *EMG* electromyography

^*^significant difference, **significant difference in subgroup, †not significant

**Fig. 2:**
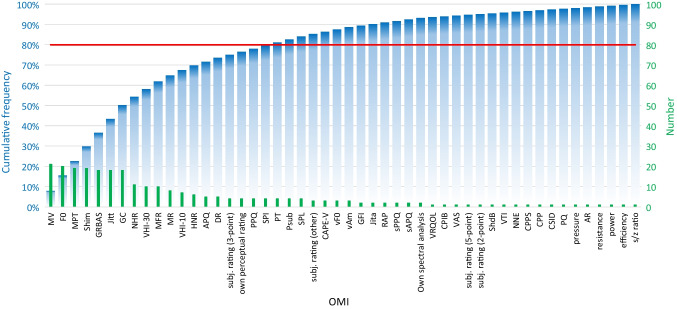
Pareto diagram of OMIs**.** *MV* mucosal wave, *F0* fundamental frequency, *MPT* maximum phonation time, *Shim* shimmer, *GRBAS* grade roughness breathiness asthenic strain, *Jitt* jitter, *GC* glottic closure, *NHR* noise to harmonic tatio, *VHI* voice handicap index, *MFR* mean flow rate, *MR* melodic range, *HNR* harmonic to noise ratio, *APQ* amplitude pertubation quotient, *DR* dynamic range, *PPQ* pitch perturbation quotient, *SPI* soft phonation index, *PT* phonation time, *Psub* subglottal pressure, *SPL* sound pressure level, *CAPE-V* consensus auditory-perceptual evaluation of voice, *vF0* fundamental frequency coefficient variation, *vAm* peak-to-peak amplitude, *GFI* glottal function index, *Jita* absolute jitter, *RAP* relative average perturbation, *sPPQ* smoothed pitch perturbation quotient, *sAPQ* smoothed amplitude pertubation quotient, *VRQOL* voice related quality of life, *CPIB* communicative participation item bank, *VAS* visual analog scale, *ShdB* absolute shimmer, *VTI* voice turbulence index, *NNE* normalized noise energy, *CPPS* smoothed cepstral peak prominence, *CPP* cepstral peak prominence, *CSID* cepstral spectral index of dysphonia, *PQ* phonation quotient, *AR* aerodynamic resistance

A total of 50 different OMIs were reported in the 34 selected studies. With most OMIs being used in multiple studies the total number of OMIs reported was 265. The frequencies of the different OMIs are shown in Table 3 and additionally as a Pareto diagram in Fig. 2. Nineteen (19) OMIs accounted for 80% of reports. Table 4 shows an overview of these top 19 OMIs identified from the Pareto diagram divided into the ELS subcategories, together with the percentage of studies that find a significant impact after treatment. Five of these OMIs show a significant change after treatment in more than half of the studies that reported on them.

**Table 3:** Frequency of OMIs, overall percentage (%), cumulative percentage (%)

OMI	Frequency	Percentage (%)	Cumulative
			Percentage (%)
MV	21	7,9%	7,9%
F0	20	7,5%	15,5%
MPT	19	7,2%	22,6%
Shim	19	7,2%	29,8%
GRBAS	18	6,8%	36,6%
Jitt	18	6,8%	43,4%
GC	18	6,8%	50,2%
NHR	11	4,2%	54,3%
VHI-30	10	3,8%	58,1%
MFR	10	3,8%	61,9%
MR	8	3,0%	64,9%
VHI-10	7	2,6%	67,5%
HNR	6	2,3%	69,8%
APQ	5	1,9%	71,7%
DR	5	1,9%	73,6%
subj. rating (3-point)	4	1,5%	75,1%
own perceptual rating	4	1,5%	76,6%
PPQ	4	1,5%	78,1%
SPI	4	1,5%	79,6%
PT	4	1,5%	81,1%
Psub	4	1,5%	82,6%
SPL	4	1,5%	84,2%
subj. rating (other)	3	1,1%	85,3%
CAPE-V	3	1,1%	86,4%
vF0	3	1,1%	87,5%
vAm	3	1,1%	88,7%
GFI	2	0,8%	89,4%
Jita	2	0,8%	90,2%
RAP	2	0,8%	90,9%
sPPQ	2	0,8%	91,7%
sAPQ	2	0,8%	92,5%
own spectral analysis	2	0,8%	93,2%
VRQOL	1	0,4%	93,6%
CPIB	1	0,4%	94,0%
VAS	1	0,4%	94,3%
subj. rating (5-point)	1	0,4%	94,7%
subj. rating (2-point)	1	0,4%	95,1%
ShdB	1	0,4%	95,5%
VTI	1	0,4%	95,8%
NNE	1	0,4%	96,2%
CPPS	1	0,4%	96,6%
CPP	1	0,4%	97,0%
CSID	1	0,4%	97,4%
PQ	1	0,4%	97,7%
pressure	1	0,4%	98,1%
AR	1	0,4%	98,5%
resistance	1	0,4%	98,9%
power	1	0,4%	99,2%
efficiency	1	0,4%	99,6%
s/z ratio	1	0,4%	100,0%
	265	100	

*MV* mucosal wave, *F0* fundamental frequency, *MPT* maximum phonation time, *Shim* shimmer, *GRBAS* grade roughness breathiness asthenic strain, *Jitt* jitter, *GC* glottic closure, *NHR* noise to harmonic tatio, *VHI* voice handicap index, *MFR* mean flow rate, *MR* melodic range, *HNR* harmonic to noise ratio, *APQ* amplitude pertubation quotient, *DR* dynamic range, *PPQ* pitch perturbation quotient, *SPI* soft phonation index, *PT* phonation time, *Psub* subglottal pressure, *SPL* sound pressure level, *CAPE-V* consensus auditory-perceptual evaluation of voice, *vF0* fundamental frequency coefficient variation, *vAm* peak-to-peak amplitude, *GFI* glottal function index, *Jita* absolute jitter, *RAP* relative average perturbation, *sPPQ* smoothed pitch perturbation quotient, *sAPQ* smoothed amplitude pertubation quotient, *VRQOL* voice related quality of life, *CPIB* communicative participation item bank, *VAS* visual analog scale, *ShdB* absolute shimmer, *VTI* voice turbulence index, *NNE* normalized noise energy, *CPPS* smoothed cepstral peak prominence, *CPP* cepstral peak prominence, *CSID* cepstral spectral index of dysphonia, *PQ* phonation quotient, *AR* aerodynamic resistance

**Table 4:** Percentage of studies per OMI showing significant results between pre- and post-treatment

	OMI	*p* value ≤ 0.05	Percentage of significance (%)
Subjective	VHI-30	9/10 (1 NS,—NA)	90.0*
	VHI-10	4/7 (- NS, 3 NA)	57.1*
	subjective rating (3-point)	1/4 (- NS, 3 NA)	25.0
Perceptual	GRBAS	13/18 (2 NS, 3 NA)	72.2*
	own perceptual rating	1/4 (-NS, 3 NA)	25.0
Acoustic	F0	6/20 (10 NS, 4 NA)	30.0
	Shimmer	6/19 (11NS, 2 NA)	31.6
	Jitter	8/18 (8 NS, 2 NA)	44.4
	NHR	5/11 (4 NS, 2 NA)	45.5
	HNR	0/6 (5 NS, 1 NA)	0.0
	MR	4/8 (2 NS, 2NA)	50.0
	APQ	3/5 (1 NS, 1NA)	60.0*
	PPQ	2/4 (1 NS, 1NA)	50.0
	SPI	0/4 (3 NS, 1 NA)	0.0
Aerodynamic	MPT	13/19 (5 NS, 1 NA)	68.4*
	MFR	4/10 (4 NS, 2 NA)	40.0
	DR	1/5 (4 NS,—NA)	20.0
Endoscopic	MV	10/21 (1 NS, 10 NA)	47.1
	GC	5/18 (1 NS, 12 NA)	27.8

*VHI* voice handicap index, *GRBAS* grade roughness breathiness asthenic strain, *F0* fundamental frequency, *NHR* noise to harmonic ratio, *HNR* harmonic to noise ratio, *MR* melodic range, *APQ* amplitude pertubation quotient, *PPQ* pitch perturbation quotient, *SPI* soft phonation index, *MPT* maximum phonation time, *MFR* mean flow rate, *DR* dynamic range, *MV* mucosal wave, *GC* glottic closure

*NS* not significant, *NA* not available

^*^ > 50% “percentage of significance

## Discussion

In this review we identified the OMIs most used to evaluate treatment effect in patients with non-paralytic glottic insufficiency caused by vocal fold atrophy with and without sulcus. A total of 50 different OMIs were identified with 19 of these accounting for 80% of total reported OMIs. Of these 19 OMIs, five showed a significant change after treatment in more than half of the studies where they were used.

Interestingly, of the top ten most used parameters most were acoustic, aerodymamic or stroboscopic. Only one patients’ self-evaluation parameter was included in the top ten which was the VHI-30 ranked as 9th most used while self-evaluation is one of the most clinically relevant tools for measuring treatment outcome in daily practice. Additionally, several studies have proposed that it is the one most reliable tool for evaluating treatment response in this patient population [2, 45, 46]. This review shows it has a much higher percentage of significance than the acoustic or endoscopic parameters.

It is well known that assessing voice outcome after treatment is complex and that multidimensional evaluation is necessary. With the large body of OMIs available, choosing representative and reliable parameters is challenging and much evidence points to the fact that disease specific core outcome sets of OMIs are needed [10]. Before formulating such a COS a basic overview of parameters used in literature is required. It is important to emphasize that the parameters found to be the most frequently used for patients with non-paralytic glottic insufficiency caused by vocal fold atrophy with or without sulcus in this review may not necessarily be the most appropriate for this cohort. To properly assess the usefulness of an OMI, not only frequency of use, but also clinical relevance, applicability and psychometric validity are important factors to consider [10].

However, as a starting point, it is valuable to have insight into the choices that are currently being made by clinicians. The findings of this review can guide further initiatives on the route to a COS by indicating which parameters should be prioritized going forwards. The top OMIs revealed by this review as well as the factors for determining the ultimate relevance of an OMI are discussed below.

### Subjective OMIs

The VHI-30 was the most frequently used subjective OMI (n = 10, 9th rank) and had a very high percentage of significance at 90%. The VHI-10 was the second most used (n = 7, 12th rank) with a lower percentage of significance of 57%. Around 75% of the studies in this review (26 out of 34) used a form of subjective rating. Subjective evaluation is one of the most clinically relevant tools in the communication with patients. The VHI is a robust questionnaire with translations and validations in many languages and it has the most sufficient psychometric construct based on COSMIN taxonomy [47, 48]. One may argue that VHI is designed for dysphonic patients in general and not specifically for patients with glottic insufficiency and that a questionnaire especially designed for glottic insufficiency may be preferred above VHI. More focused questionnaires for a future COS could be the glottal function index (GFI)[49], vocal fatigue index (VFI)[50] or vocal fatigue handicap questionnaire (VFHQ)[51] with the GFI having the advantage above the other disease specific questionnaires being the only OMI with moderate positive rating on psychometric ratings [48].

However, it is also important to consider that instead of incorporating ever more detailed disease specific PROMs (Patient-reported Outcome Measurement), there is also a countercurrent in literature supporting the development and use of generic PROMs focusing on general health aspects such as physical, mental and social health including quality of sleep or ability to work. An initiative to develop and measure generic PROMs is PROMIS (Patient-reported Outcomes Measurement Information System) which is an innovative, intelligent system for measuring generic PROMs to be used for different health problems and diseases (www.healthmeasures.net). A generic, non-disease specific health survey may be also of interest as a quality of life measurement instrument which can be used for cost-utility analysis by measuring quality-adjusted life years (QALYs) such as the EQ-5d (EuroQol 5D) [52].

### Perceptual OMIs

The GRBAS was the most frequently used perceptual OMI (n = 18, 5th rank), with a percentage of significance of 72%. GRBAS is a widely used perception scale. The G, general grade, has a satisfactory inter- and intra-rater reliability and is therefore suitable as a single OMI. In the latest ELS proposal the use of complete GRBAS scale is preferred [4]. A main disadvantage of using perceptual OMIs in patients with non-paralytic glottic insufficiency is that structural defects, such as sulcus, are not always addressed in treatments, such as medialization procedures, where the primary goal of treatment is to improve endurance and not perceptual quality of the voice [17, 22].

### Acoustic OMIs

Interestingly, our review showed that studies relied heavily on acoustic OMIs such as fundamental frequency (F0) (n = 20, 2nd rank), shimmer (n = 19, 4th rank), jitter (n = 18, 6th rank) and noise to harmonic ratio (NHR) (n = 11, 8th rank), even though none of these acoustic parameters achieved a percentage of significance above 50%. Their high frequency of use is likely due to them being automatically provided by most voice programs, but their clinical usefulness may be less defined. They are less intuitive in communications with patients, in our and other’s experience, and have been shown not to correspond to more clinically relevant parameters [2, 17, 22, 45].

Nevertheless, acoustic OMIs could potentially aid in detecting differences in the regularity of phonation that may be missed with more broad spanning parameters such as perceptual evaluation. The challenge would be to find the appropriate ones for this specific patient population from the large number of parameters available. Despite its low ranking and lack of significance in our review, one example could be the soft phonation index (SPI) (n = 4, 19th rank, 0% percentage of significance) which reflects the approximation of vocal folds [53]. It’s possible usefulness has been shown in unilateral nodules, but, to our knowledge, has not been clarified in atrophy and/or sulcus [54]. Inconsistency in normal values and increased SPI for pressed phonation have been seen [53, 54]. This may hamper the interpretation of SPI in atrophy and sulcus.

### Aerodynamic OMIs

Of the top 19 OMIs used, three were aerodynamic; maximum phonation time (MPT) (n = 19, 3th rank), mean flow rate (MFR) (n = 10, 10th rank), and dynamic range (DR) (n = 5, 15th rank). MPT is a well-known voice parameter; it is simple, reliably obtainable, but with the disadvantage that normative data will differ in sub-populations depending on gender or age [55, 56]. MPT has been found to be the most used and most significant OMI for UFVP (90% percentage of significance) [5]. Our results indicated a less prominent role in our patient group (68% percentage of significance), possibly due to the difference in underlying pathology, including the degree of glottal gap that needs correction. Aerodynamic OMIs that require a pneumotachograph are less easy to obtain, f.e. MFR or phonation quotient ((PQ)(vital capacity/MPT)) as alternative. MFR may be of value for glottic insufficiency with mobile vocal folds, as it is for immobile vocal fold in UVFP, stated by Desuter et al., with relatively high ranking and percentage of significance (86% percentage of significance) [5].

Another measurement of interest is the phonation threshold pressure (Pth). It reflects the minimum subglottic pressure needed to reach phonation onset and sustain phonation [57]. It may be more appropriate to capture the subtle changes in subglottic pressure when comparing pre- and posttreatment effect. It has found only limited use up till now, although a preliminary study in 2021 showed that measuring Pth in UVFP is feasible [58]. Attributing factors for this may be variations in procedural methodology for task elicitation as well as environmental and participant inconsistencies that might affect phonation threshold pressure values [59].

### Endoscopic OMIs

Mucosal wave was the most used OMI (n = 21, 1th rank) followed by glottic closure (n = 18, 7th rank), although both had a relative low percentage of significance (47% and 28% respectively). It is therefore debatable if endoscopic parameters are the most suitable OMIs for this patient population due to the inherent inter-observer bias associated with this form of assessment and the combined pathology of atrophy and sulcus leading to further difficulties in assessing exams [4, 60].

However, as endoscopy is broadly used in this patient group, more systematic and detailed videolaryngostroboscopic assessment protocols should be investigated, f.e. as described in VALI (Voice-Vibratory Assessment with Laryngeal Imaging)[61]. Frame-by-frame- analysis (FBFA) could also be useful [62]. Another possibility would be to use disease specific laryngoscopic assessments. For vocal fold atrophy, the reliability of laryngoscopic features have been investigated with satisfying results and recently a validated classification of presbylarynx based on laryngoscopic findings has been published [63, 64].

As stated in the introduction, to properly assess the usefulness of an OMI, before it can be included in a COS, quality assessment has to be performed. In doing so, not only frequency of use, but also clinical relevance, applicability and psychometric validity are important factors to consider [10].

To address the issue of the relevance we calculated the “percentage of significance” for the most frequently used OMIs, defined by Desuter et al. as the percentage of number of studies with a significant change in a specific OMI, divided by total number of studies using this OMI [5]. We found the VHI-30 to be the only OMI with a percentage of significance higher than 80% and the VHI-10, GRBAS, MPT and the APQ to be the only parameters of 50% or more. Interestingly, Desuter et al. found percentages of significance higher than 80% for MPT (90%), mean airflow (86%) and the G of the GRBAS (85%) in his review on unilateral vocal fold paralysis. We hypothesize that this difference may reflect the pathophysiological difference between glottic insufficiency with mobile vocal folds and UVFP, supporting the notion that the relevance of OMIs may differ from disease to disease.

Studies tend to report mainly on the statistical significance of a change in an OMI, which does not necessarily correspond to a difference that is clinically relevant. But for patients and health professionals clinically relevant changes in outcome are of great importance.

Until now, the clinical relevance of a certain outcome has often been consensus based [31]. However, values for clinically relevant changes have been suggested for some of these OMIs. Van Gogh et al. defined what constitutes a clinically relevant change for the VHI-30 based on a selected Dutch population with dysphonia after treatment for early glottic cancer or benign voice disorders and a normal population [65]. More recently Young et al. formulated the MCID (minimal clinically important difference) for VHI-10 in patients with vocal fold paralysis. The authors highlight that not only the numerical change within a parameter that represents a minimal clinically relevant change is important, but also that this value may be disease specific [66]. Therefore, some OMIs may not be as valuable for a specific disease as traditionally assumed.

Applicability, whether a test can be performed or not, depends on logistic, technical and financial possibilities and limitations. For acoustic, aerodynamic, but also endoscopic OMIs this can be a limiting factor. For acoustic measurements special voice program software is needed to record and store a phonetogram, and to extract, calculate and store various voice parameters. These programs are commercially available, f.e. MDVP (multidimensional voice program software, computerized speech laboratory (KayPENTAX, Montvale, NJ)) and have their own set of parameters. For aerodynamic parameters as MFR a pneumotachograph is needed (phonatory aerodynamic system (PAS), KayPENTAX, Montvale, NJ).

The last important factor is psychometric validity. Psychometric validity has been only investigated for subjective OMIs [48, 67]. In the study of Francis et al. 32 PROMs were reviewed on development and validation and showed gross psychometric weaknesses as lack of patient involvement, lack of robust construct validity and lack of interpretability and scaling [67]. Speyer et al. reported on psychometric properties of 15 PROMs and concluded that many psychometric data were missing or indeterminate, VHI seeming to be the most promising questionnaire [48].

This study has some several weaknesses. First of all, no formal Risk of Bias (RoB) was performed. We found this of limited added value, because most studies, 32 out of 34 were cohort studies of which 25 retrospective, with a comparable risk of bias. Of the 2 clinical trials, there was only one double blind RCT, which has a low RoB. Secondly, no formal meta-analysis was performed. As statically significance does not always correspondent with clinically relevancy we chose “percentage of significance” to capture relevancy, although this may not be the most thorough way of doing this. Lastly, we would like to emphasize that the most frequently used OMIs, collected in this review, do not defacto represent the most appropriate OMIs for this patient group, and that besides frequency of use, also clinical relevance, applicability, and psychometric validity are important factors to consider.

## Conclusion

In this systematic review we identified the most used OMIs to evaluate treatment effect in patients with non-paralytic glottic insufficiency caused by vocal fold atrophy with and without sulcus as a second step towards developing a COS for this population. The need for a COS is further demonstrated by the fact that studies in this review rely heavily on parameters that have a low percentage of significance in this population, with the exception of VHI-30 with a high percentage of significance of 90%. Future steps in this process will include a quality analysis of the identified OMIs for this specific use and final inclusion through a Delphi process.
